# Performances of Targeted RNA Sequencing for the Analysis of Fusion Transcripts, Gene Mutation, and Expression in Hematological Malignancies

**DOI:** 10.1097/HS9.0000000000000522

**Published:** 2021-01-27

**Authors:** Sandrine Hayette, Béatrice Grange, Maxime Vallee, Claire Bardel, Sarah Huet, Isabelle Mosnier, Kaddour Chabane, Thomas Simonet, Marie Balsat, Maël Heiblig, Isabelle Tigaud, Franck E. Nicolini, Sylvain Mareschal, Gilles Salles, Pierre Sujobert

**Affiliations:** 1 Hospices Civils de Lyon, Hôpital Lyon Sud, Service d’hématologie biologique, Pierre-Bénite, France; 2 Cancer Research Center of Lyon, INSERM U1052 UMR CNRS 5286, Equipe labellisée Ligue Contre le Cancer, Université de Lyon, France; 3 French group of CML, Centre Léon Bérard, Lyon, France; 4 Hospices Civils de Lyon, Plateforme de séquençage NGS-HCL, cellule bioinformatique, Bron, France; 5 Université de Lyon, Université Lyon 1, CNRS, Laboratoire de Biométrie et Biologie Evolutive UMR 5558, Lyon, France; 6 Hospices Civils de Lyon, Hôpital Lyon Sud, Service d’hématologie clinique, Pierre-Bénite, France; 7 Hematology Department and INSERM U1052, CRCL, Centre Léon Bérard, Lyon, France.

## Abstract

RNA sequencing holds great promise to improve the diagnostic of hematological malignancies, because this technique enables to detect fusion transcripts, to look for somatic mutations in oncogenes, and to capture transcriptomic signatures of nosological entities. However, the analytical performances of targeted RNA sequencing have not been extensively described in diagnostic samples. Using a targeted panel of 1385 cancer-related genes in a series of 100 diagnosis samples and 8 controls, we detected all the already known fusion transcripts and also discovered unknown and/or unsuspected fusion transcripts in 12 samples. Regarding the analysis of transcriptomic profiles, we show that targeted RNA sequencing is performant to discriminate acute lymphoblastic leukemia entities driven by different oncogenic translocations. Additionally, we show that 86% of the mutations identified at the DNA level are also detectable at the messenger RNA (mRNA) level, except for nonsense mutations that are subjected to mRNA decay. We conclude that targeted RNA sequencing might improve the diagnosis of hematological malignancies. Standardization of the preanalytical steps and further refinements of the panel design and of the bioinformatical pipelines will be an important step towards its use in standard diagnostic procedures.

## Introduction

In hematological malignancies as in cancer in general, the goal of the diagnosis procedures is not only to accurately classify the patient’s disease according to the consensual World Health Organization guidelines,^1^ but also to identify biomarkers of prognostic or predictive values. A part of this information can be captured by morphology and immunophenotyping, but it relies more and more on the analysis of the genomic alterations of the neoplastic cells.^2^ Nowadays, conventional cytogenetics and targeted sequencing of relevant genes are still the standard procedures. However, technological outbreaks such as whole genome sequencing, assay for transposase-accessible chromatin-sequencing, or RNA sequencing (RNA-seq) might refine the diagnosis by unraveling genomic alterations outside coding regions,^3^ epigenetic signatures,^4,5^ and gene expression profiles, respectively.^6^

In this study, we have chosen to assess the diagnostic value of RNA-seq, because this technique allows to explore 3 levels of genetic information: gene sequence, gene fusions, and gene expression. Interestingly, each of these different levels of analysis brings independent information about the neoplastic cell, and accordingly, their integration should refine the precision of the diagnosis. For example, acute myeloid leukemia (AML) patients prognosis is evaluated by cytogenetics (copy number abnormalities and structural variants), further refined by the analysis of the mutational status of a few genes, and could maybe be improved by transcriptomic signatures such as the 17-gene leukemia stem cell score (LSC17) which is a proxy of the number of leukemic stem cells.^7^

Different techniques of library preparation for RNA-seq have been described, enabling the analysis of all the RNA molecules of a sample, or using enrichment step to target genes of interest such as messenger RNA, or small RNA species. Of note, the choice of the library preparation should optimize the balance between the number of targets of interest and the required depth of sequencing, in order to remain economically affordable in a routine setting. To date, most of the genes involved in cancer have been already identified by large programs of whole exome sequencing.^8^ Based on these considerations, we have decided to evaluate the performances of a targeted RNA-seq panel of 1385 genes involved in cancer biology. We present here the analytical performances of targeted RNA-seq to detect fusion transcripts, to identify transcriptional profiles associated with clinically relevant entities, and to detect the recurrent mutations with clinical significance in hematological malignancies.

## Materials and methods

### Samples

One hundred diagnosis samples from patients with the following hematological malignancies were included as follows: acute leukemia (AML, n = 51 including 7 acute promyelocytic leukemia [APL], B-cell acute lymphoblastic leukemia [ALL] [n  = 27], mixed phenotype acute leukemia [n = 1], and T-cell ALL [n = 1]); myeloproliferative neoplasms (chronic myeloid leukemia [CML, n = 12] and other myeloproliferative neoplasms [n = 2]); hypereosinophilic syndromes (HESs, n = 3); chronic myelomonocytic leukemia (CMML, n = 2); and myelodysplastic syndrome with multilineage dysplasia (n = 1). These samples were chosen to enrich the cohort in fusion transcript due to chromosomal translocations, based on the results of conventional cytogenetics, in order to test the performances of targeted RNA-seq to detect fusion transcripts. Moreover, we used 4 controls (C1 to C4) prepared by pooling blood samples from 5 healthy donors for each, and 4 bone marrow samples from healthy donors. The characteristics of the samples are provided in Supplemental Table 1 (http://links.lww.com/HS/A125). The procedures followed were in accordance with the Helsinki Declaration, as revised in 2008.

Cytogenetic R and G-banding analyses were performed according to standard methods. The definition of a cytogenetic clone and description of karyotypes followed the current International System for Human Cytogenetic Nomenclature.

For a subset of samples (n = 45), the analysis of a panel of 105 genes was already performed for routine diagnostic procedures, as already described.^9^

### RNA extraction

Three different protocols of RNA extraction were used (Supplemental Table 1, http://links.lww.com/HS/A125). For ALL samples and 3 bone marrow samples from healthy donors, RNA was extracted with Trizol reagent (TRIZ: Invitrogen, Carlsbad, California). For AML samples, RNA was extracted with NucleoSpin RNA kit (MN: Macherey Nagel, Düren, Germany). For CML, HES, CMML, and myelodysplastic syndrome samples, RNA was extracted with MN or the Maxwell 16 LEV simplyRNA Blood Kit (Max: Promega, Madison, Wisconsin). For control samples C1 to C4, RNA was extracted after Ficoll enrichment with either Trizol or MN methods, in order to assess the effect of extraction protocol on transcriptomic analysis performances. RNA quality was assesses by reverse transcription quantitative polymerase chain reaction (RT-qPCR) of the ABL1 messenger RNA (mRNA), which was always above 32 000 copies.

### RNA sequencing

Library preparation was performed from 20 ng of RNA using TruSight RNA Pan-Cancer Panel (Illumina, SanDiego, California) targeting 1385 genes involved in cancer biology (panel available at https://www.illumina.com/content/dam/illumina-marketing/documents/products/gene_lists/gene_list_trusight_pan_cancer.xlsx). Libraries from 16 samples were multiplexed and sequenced on a Nextseq 500 device (Illumina) with a 2 × 81 paired-end run on a mid-output flowcell according to the manufacturer’s instructions (mean number of reads by sample: 32 × 10^6^; range 20–59 × 10^6^).

### Bioinformatical analysis

After demultiplexing, adapter sequences were trimmed with Cutadapt and reads were mapped to the human genome (Genome Reference Consortium Human Build 37). The percent of reads aligned to ribosomal RNA determined with the RSeqC software was around 0.25% of the total reads before filtering on the bed. The detection of gene fusions was performed first with the commonly used STAR-Fusion pipeline (parameters: FusionInspector validate) and STAR-2pass,^10^ and all the negative samples were reanalyzed with the recently launched nf-core^11^ and Arriba (https://github.com/suhrig/arriba/) pipelines. Putative fusions were validated by reverse transcription and polymerase chain reaction (primers sequences are provided in Supplemental Table 2, http://links.lww.com/HS/A126). Gene expression analysis (after trimmed mean of M values normalization), principal component analysis, k-means clustering, 2-tailed *t*-test, and Heat Map generation followed by hierarchical clustering were performed using Omics Explorer software (Qlucore AB, Lund, Sweden). For gene mutation analysis on RNA-seq data, we looked at all the mutations found at the DNA level by combining the same homemade workflow as for DNA and visual inspection of the binary alignment map files in case of unfound mutation. In brief, we first gather the variant alleles called with Freebayes and Varscan2.^12,13^ Among this raw set, we kept alleles whose read frequency was either above 20% or for those below, if their frequency was more than 5-fold the median of the frequencies of all the samples from the same run. A second filtering step was applied to get rid of variants whose occurrence was above 1% in Genome Aggregation Database mixed populations.^14^

## Results

### Identification of fusion transcripts

**Figure 1.**
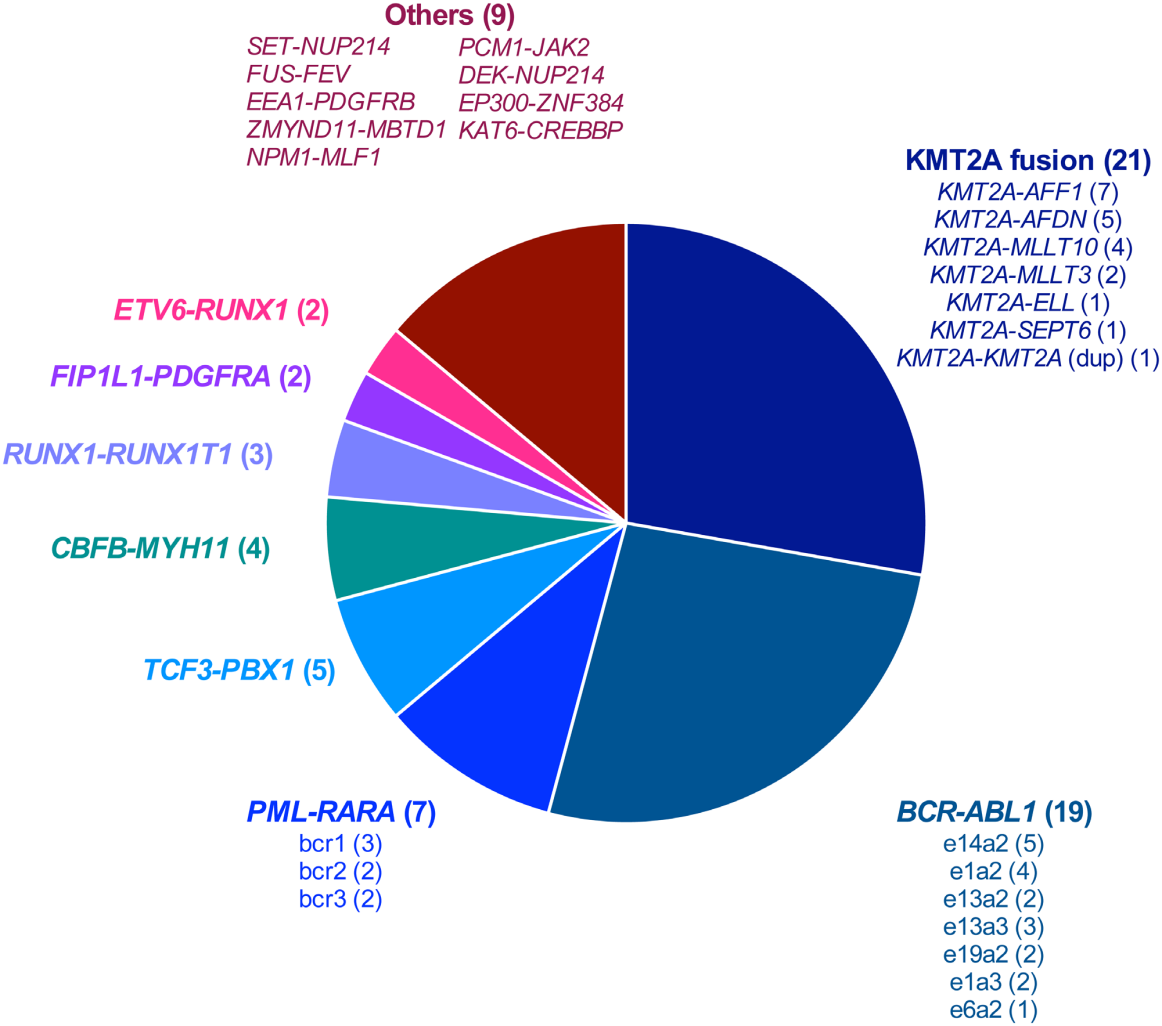
**Description of the 72 fusion transcripts detected by targeted RNA-seq in the whole cohort.** RNA-seq = RNA sequencing.

Fusion transcript positivity threshold was determined by the detection of at least 1 junction read and 1 spanning read between 2 different genes. All putative new fusion transcripts have been validated by PCR. All of the 57 rearrangements identified by cytogenetics or molecular biology were identified by targeted RNA-seq (Figure 1). Notably, RNA-seq detected all the *BCR-ABL1* canonical and rare transcripts (e13a2 [n = 2]; e14a2 [n = 5]; e1a2 [n = 4]; e1a3 [n = 2]; e6a2 [n = 1]; e13a3 [n = 3]; and e19a2 [n = 2]), as well as all the *PML-RARA* transcripts (BCR1 [n = 2] BCR2 [n = 2]; BCR3 [n = 3]) and *MLL* (*KMT2A*) fusions (n = 19). Of note, 2 samples with *FIP1L1-PDGFRA* fusion transcripts and 1 with *KMT2A* duplication were missed when analyzed with the STAR-fusion pipeline but recovered with nf-core and Arriba bioinformatics pipelines.

**Figure 2.**
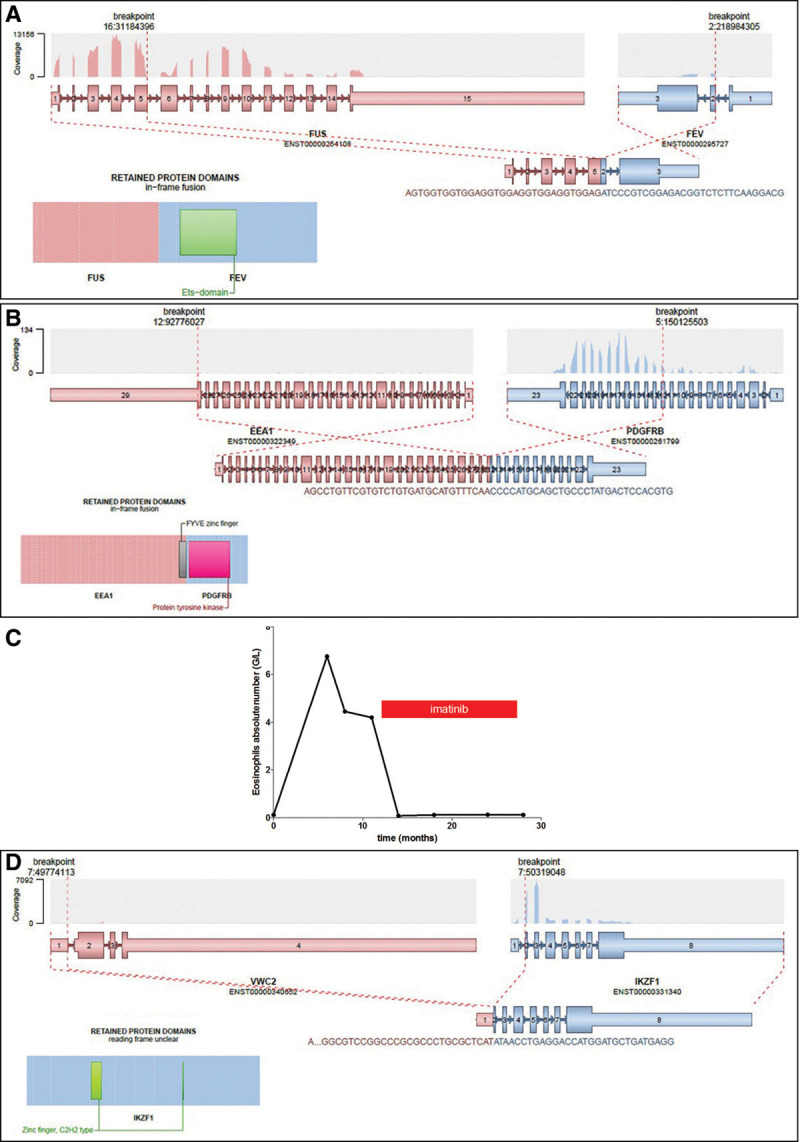
**Description of the 3 new fusion transcripts discovered in this cohort.** Schematic representation of the 3 new fusion transcripts identified by targeted RNA-seq: *FUS-FEV* from t(2;16) (A), *EEA1-PDGFRB* from t(5;12) (B), evolution of the eosinophil count of the platelet with the EEA1-PDGFRB fusion transcript under imatinib treatment (C), and *VWC2-IKZF1* (D). For each fusion, transcript is provided a schematic representation of the translocation at the genomic level, a graphical representation of the coverage depth in the targeted RNA-seq, and a schematic representation of the protein fusion. RNA-seq = RNA sequencing.

Eighteen samples had a chromosomal translocation without detected fusion transcript based on routine molecular biology tests, which are designed to detect recurrent fusion transcripts. Targeted RNA-seq did not find any fusion transcript in 11 samples. In 5 patients, targeted RNA-seq identified a fusion transcript already described in the literature (*KAT6-CREBP*, *NPM1-MLF1*, *PCM1-JAK2*, *DEK-NUP214*, *ZMYND11-MBTD1*^15^) (Figure 1). In 2 patients, a fusion transcript never described in the literature was identified and confirmed by RT-PCR and Sanger sequencing (*FUS-FEV*; *EEA1-PDGFRB*). These fusion transcripts were in frame, probably leading to the expression of an abnormal fusion protein (Figure 2A, B). Interestingly, the patient with the EEA1-PDGFRB transcript fusion was suffering from a HES with skin lesions and splenomegaly, which fully resolved after imatinib initiation (Figure 2C).

Finally, we detected a fusion transcript in 5 samples without detectable translocation on conventional cytogenetics: *SET-NUP214*, *EP300-ZNF384*, *KMT2A-MLLT4*, *KMT2A-MLLT10*, *VWC2-IKZF1* (Figure 1). The *VWC2-IKZF1* fusion transcript (Figure 2D), never described so far, was detected in an ALL with a t(9;22) leading to the expression of the *BCR-ABL1*-transcript (patient 10, Supplemental Table 1, http://links.lww.com/HS/A125). We hypothesize that this fusion might represent a new mechanism of *IKZF1* gene inactivation recurrently identified in Phi+-ALL.^16,17^

As it was previously described in noncancer tissues and cells,^18,19^ several fusions with open reading frame were also detected in control and patients’ samples. Some of them, such as *TFG-GPR128*, *POLE-FUS*, or *OAZ1-DOT1*, were expressed at high level and have been also validated by RT-PCR and sequencing.

Finally, in order to assess the sensitivity threshold of RNA-seq to detect fusion transcripts, we analyzed serial dilutions of 2 patients with *PML-RARA* and *BCR-ABL* fusion transcripts, respectively. The detection threshold was below 6% for both fusion transcripts.

### Transcriptome analysis

The analysis of transcriptome in the routine diagnosis procedure is technically challenging, because of interferences linked to the source of the samples analyzed (e.g., bone marrow versus peripheral blood), the preparation of the samples (isolation of the mononucleated cells with Ficoll or not), the RNA extraction method, and the batch effect of library preparation and sequencing. Instead were developed signatures based on a limited number of transcripts analyzed with technical platforms such as reverse transcription multiplex ligation-dependent probe amplification^20^ or Nanostring technology.^7,21^ Here, we assessed the feasibility of transcriptome analysis based on RNA-seq of a panel of 1385 genes.

**Figure 3.**
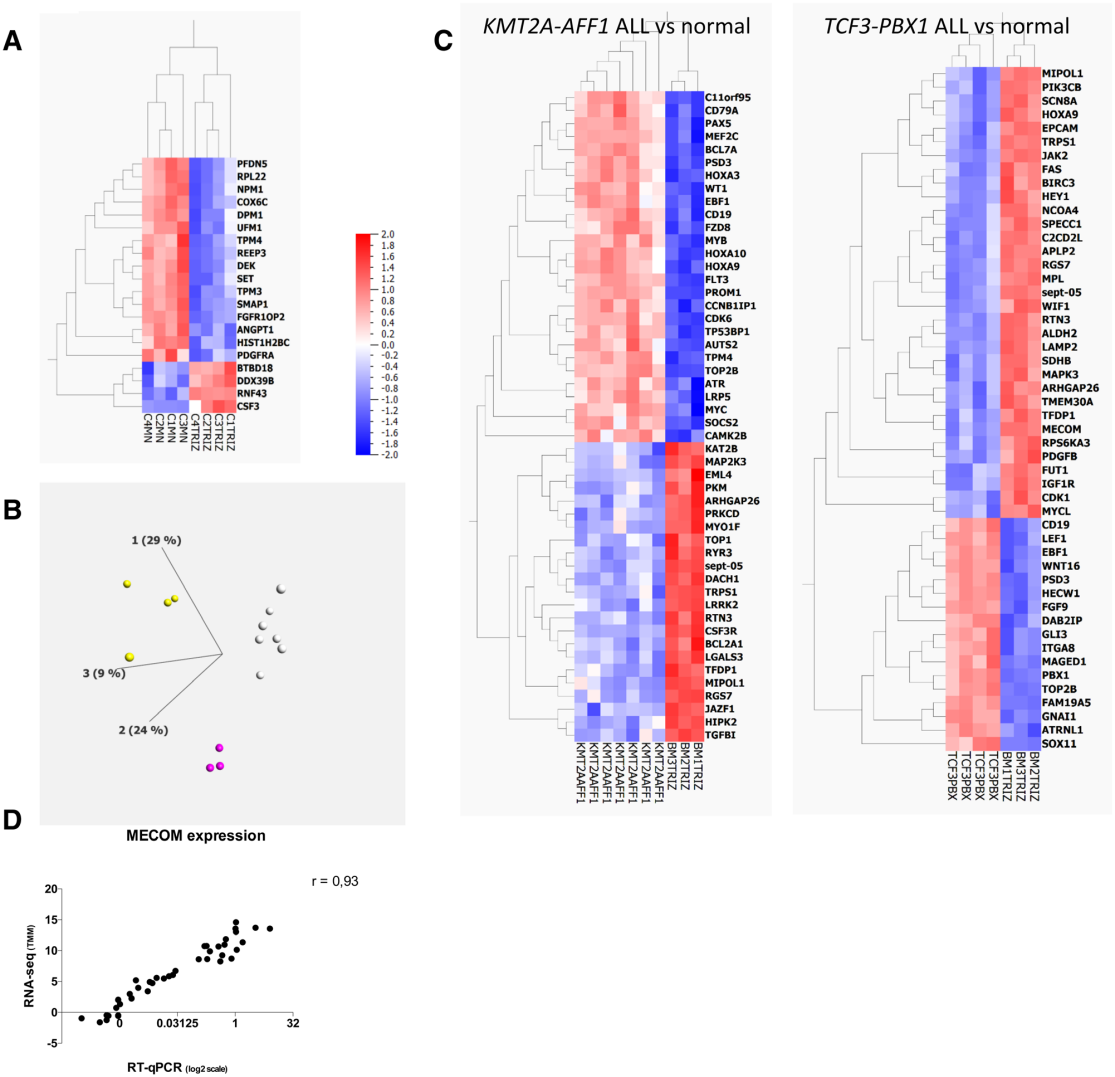
**Transcriptomic analysis of targeted RNA-seq data**. (A), Heatmap representation of the 20 genes differentially expressed (fold change > 2, q < 0.05) between the same control blood samples after RNA extraction with 2 different methods (Trizol vs Macherey Nagel). (B), Principal component analysis and unsupervised k-means clustering of 14 samples processed with the same preanalytical steps (*KMT2A-AFF1* ALL, white dots, n = 7, *TCF3-PBX1* ALL, yellow dots, n = 4, normal bone marrow samples, purple dots, n = 3). (C), Heatmaps showing the 50 most differentially expressed genes between normal samples and *KMT2A-AFF1* ALL samples (left) and between normal samples and *TCF3-PBX1* ALL samples (right). ALL = acute lymphoblastic leukemia; MECOM = MDS1 and EVI1 complex locus; RNA-seq = RNA sequencing; RT-qPCR = reverse transcription quantitative polymerase chain reaction; TMM = trimmed mean of M values.

First, we evaluated the magnitude of systematic biases in transcriptomic analysis introduced by the protocol of RNA extraction and the sequencing process. The same blood samples from healthy donors were extracted after Ficoll enrichment either with Trizol (n = 4) or with Macherey Nagel kits (n = 4). A supervised analysis based on extraction method identified 20 differentially expressed genes (fold-change threshold 2, false discovery rate q < 0.05) (Figure 3A). On the contrary, when we compared the transcriptome of RNA extracted from blood samples from healthy donors, whose RNA-seq libraries and sequencing were not prepared and run the same day, there was no gene differentially expressed according to the batch of library preparation or sequencing (fold-change threshold 2, false discovery rate q < 0.05, data not shown).

Then, we analyzed bone marrow samples extracted with the same method (Trizol) from 3 groups with at least 3 patients: ALL with *KMT2A-AFF1* (n = 7), ALL with *TCF3-PBX1* (n = 4), and normal bone marrow controls (n = 3). Of note, these RNA were extracted at the time of diagnosis, over a period of 19 years, introducing a potential bias due to differences in RNA conservation. Clustering of these samples in 3 categories (by the k-means method) distinguishes the 3 groups of samples according to the diagnosis, with no misclassification (Figure 3B). The analysis of the 50 most differentially expressed genes between control and both types of ALL confirmed previously described features such as *HOXA3*, *HOXA9*, *HOXA10*, and *FLT3* overexpression in *KMT2A-AFF1*^22^ and *CD19*, *WNT16*, and *PBX1* up-regulation in *TCF3-PBX1* (Figure 3C).^23^ Gene expression is also important to decipher the prognosis of patients. For example, around 10% of AML strongly express the *MECOM* transcript, which is associated with poor prognosis. For 44 AML patients of the cohort, we compared the expression level of *MECOM* as determined by RT-qPCR and by RNA-seq. As shown in Figure 3D, we observed a strong correlation between both measures (spearman correlation r = 0.93, *P* < 0.0001), which suggests that targeted RNA-seq might also be able to evaluate prognostic signatures based on gene expression.

### Detection of gene mutations

**Figure 4.**
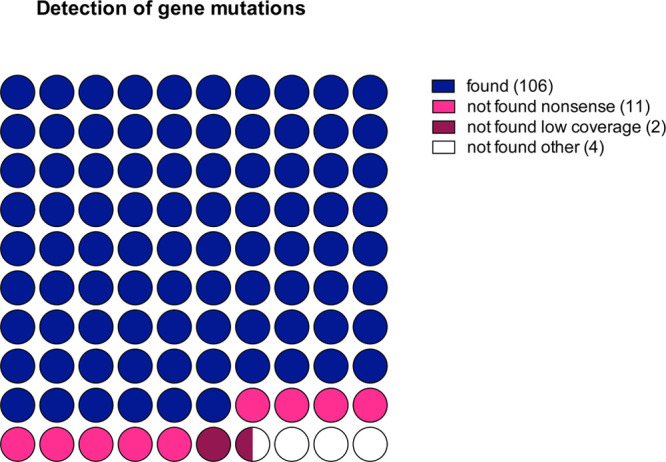
**Performances of somatic mutations detection based on RNA-seq analysis.** The relative number of mutations correctly identified or undetected (nonsense, low coverage, or other) are presented. RNA-seq = RNA sequencing.

Forty-five patients analyzed with targeted RNA-seq were also analyzed at the DNA level for a panel of 105 genes recurrently mutated in hematological malignancies.^9^ Among the 95 genes captured in both panels, 122 mutations were detected at the DNA level in 39 different genes (Supplemental Table 3, http://links.lww.com/HS/A127). As shown in Figure 4, 106 of 122 mutations (87%) identified at the DNA level were also found in the RNA-seq data. Among the 16 mutations missed at the mRNA level, frameshift mutations were overrepresented (missed mutations 11/16 versus 12/106, Fisher exact test *P* < 0.0001). Two other missed mutations (I1897T and G218V from TET2 and U2AF1, respectively) were in low coverage areas (<30×). Of note, when analyzing only the genes contained in both panels (DNA and RNA), we did not find any additional mutation on RNA-seq.

## Discussion

This work reports the analytical performances of RNA-seq of a panel of 1385 genes to improve the diagnosis of hematological malignancies, based on a series of 100 diagnosis samples and 8 controls.

Overall, this technique detect 100% of fusion transcripts of these samples, including *FIP1L1-PDGFRA* fusions, which often require nested PCR to be identified because of low levels of expression.^24^ Of note, 2 fusion transcripts were found only by alternative bioinformatics pipelines, which highlights the major impact of the bioinformatics analysis on the performances of targeted RNA-seq. This might explain suboptimal detection of *KMT2A* and *PDGFRA* fusions in previous studies.^25^ Interestingly, RNA-seq allowed the identification of 12 fusion transcripts which were not suspected with usual analysis recommended in the diagnosis of hematological malignancies.^26^ As more and more case reports describe successful opportunistic use of targeted therapies in patients with fusion transcripts,^27–29^ the identification of unexpected fusion transcripts might offer interesting targets in relapsed/refractory patients, as was the case for the patient treated with imatinib for the *EEA1-PDGFRB* fusion-driven HES. Moreover, as translocations are most of the time drivers events which are stable during disease evolution,^30^ they can be used to track minimal residual disease with high-sensitivity RT-qPCR and adapt therapeutic intensity accordingly. However, it remains to be determined if the prognostic impact of minimal residual disease described for core binding factor AML,^31^ CML, or APL is also true for the less recurrent fusion transcripts. In 11 patients with a chromosomal translocation, we did not detect a fusion transcript. We can hypothesize that these translocations contribute to oncogenesis without a fusion transcript, as is the case for the translocations involving the immunoglobulin locus in B-cell lymphomas, for example. Alternatively, these translocations might result in fusion transcripts with low expression in the bulk of the disease, being under the threshold of detection with targeted RNA-seq, or might involve 2 genes that are not included in the panel used in this study.

Regarding the analysis of the transcriptomic profile, we show that targeted transcriptome analysis can be used for nosological purposes if the preanalytical workflow is the same for the samples analyzed. Larger series are needed to precise the performances of targeted RNA-seq to resolve this task. Another interesting question would be to assess the performances of targeted RNA-seq to measure clinically relevant signatures such as the LSC17^7^ or the more recently described six-gene leukemia stem cell score of prognostic value in pediatric AML^32^ signatures in AML, but it will need an optimization of the design of the panel to capture all relevant mRNAs.

The third clinical interest of targeted RNA-seq assessed here is the detection of acquired somatic mutations. Even if most of the mutations identified at the DNA level were found in RNA-seq data, the nonsense mutations were rarely detected. This is probably at least in part due to the phenomenon of mRNA decay, which degrades preferentially truncated mRNA,^33^ and this will remain a biological limitation of RNA-seq for mutation assessment. Finally, given the growing importance of clonal architecture analysis based on variant allele frequency (VAF) deconvolution,^34^ we should keep in mind that the VAF measured at the mRNA level might not be good surrogate markers of clonal architecture, because it takes into account allelic expression bias.

Altogether, RNA-seq of a targeted panel of genes might improve the diagnosis of hematological malignancies and highlight potential therapeutic targets. Some of the limitations of this technique might be resolved with the optimization of the panel design and the bioinformatics pipelines for hematological malignancies. However, because some limitations have a biological explanation, such as poor performances to detect nonsense mutations, RNA-seq should not replace the analysis of genomic DNA but could be rather a good orthogonal method for verifying genomic mutations and a powerful complement to increase the molecular characterization of hematologic malignancies at diagnosis.

## Disclosures

The authors have no conflicts of interest to declare.

## Supplementary Material


