# Supplementary material for: Performances of Targeted RNA Sequencing for the Analysis of Fusion Transcripts, Gene Mutation, and Expression in Hematological Malignancies
Source: Hemasphere. 2021 Jan 27;5(2):e522. doi: 10.1097/HS9.0000000000000522 (PMC8051993; doi:10.1097/HS9.0000000000000522)
Supplement: Supplementary file 2 [file hs9-5-e522-s002.pdf]

**FUSIONS**	**Forward (5'-3')**	**Reverse (5'-3')**
EEA1-PDGFRB	TGCCGACAGTGTGGAAATATCTTCTGTG	GCACAAGCTGGTCCCGCGGCAGCTC
FUS-FEV	GTGCGCGGACATGGCCTCA	GTTCTTCCCGTCCTTGAAGAG
VWC2-IKZF1	GACGAGAGCGGCTTCGTGTA	GACATGTCTTGACCCTCATCAG
**NON PATHOGENIC FUSIONS**	**Forward (5'-3')**	**Reverse (5'-3')**
OAZ1-DOT1	GCCGCACCATGCCGCTCCTAAGCCT	TCTCCATAGCGAGCTTGAGATCCGG
TFG-GPR128	GATAGTTCTGACCTTTCCTTTG	GCCATTTTCCCAGGTTCCACCAT
POLE-FUS	GCGCGGATGGCGAGGCCAGCA	CTTAATAATACCAATCTGCTTGAAGTA
