# Supplementary material for: Performances of Targeted RNA Sequencing for the Analysis of Fusion Transcripts, Gene Mutation, and Expression in Hematological Malignancies
Source: Hemasphere. 2021 Jan 27;5(2):e522. doi: 10.1097/HS9.0000000000000522 (PMC8051993; doi:10.1097/HS9.0000000000000522)
Supplement: Supplementary file 3 [file hs9-5-e522-s003.pdf]

GENE	NM	Mutation	nature of the mutation	Detected by RNAseq	occurrence
ABL1	NM_005157	p.K247R	MAF<0.01	Yes	1
ABL1	NM_005157	p.S43N	MAF<0.01	Yes	1
ASXL1	NM_015338	p.G646Wfs*12	fms	No	2
ASXL1	NM_015338	p.K726fs	fms	Yes	1
ASXL2	NM_018263	p.A497T	MAF<0.01	Yes	1
ASXL2	NM_018263	p.R591Pfs*18	fms	No	1
BCOR	NM_001123385	p.P678S	SNV	No	1
BCOR	NM_001123385	p.V668*	fms	Yes	1
CBL	NM_005188	p.R585C	SNV	Yes	1
CBL	NM_005188	p.L380P	SNV	Yes	1
CBL	NM_005188	p.C404W	SNV	Yes	1
CBL	NM_005188	pR718*	fms	No	1
CEBPA	NM_001287424	del85_309	delfms	No	1
CEBPA	NM_001287424	p.P23Nfs*81	fms	No	1
c-Kit	NM_000222	del416_421	del_e8	Yes	1
c-Kit	NM_000222	p.N822K	SNV	Yes	1
c-kit	NM_000222	p.D816V	SNV	Yes	1
c-kit	NM_000222	p.D816Y	SNV	Yes	1
c-Kit	NM_000222	p.Y418_D419insLP	SNV	Yes	1
CREBBP	NM_004380	p.Y1204C	SNV	Yes	1
CREBBP	NM_004380	p.M775I	SNV	Yes	1
CREBBP	NM_004380	p.N327L	SNV	Yes	1
CSF3R	NM_156039	p.E149D	MAF<0.01	Yes	1
CSF3R	NM_156039	p.N776*	fms	Yes	1
CSF3R	NM_156039	p.R440N	SNV	Yes	1
DNMT3A	NM_175629	p.A376T	SNV	Yes	1
DNMT3A	NM_175629	p.F252V	SNV	Yes	1
DNMT3A	NM_175629	p.R882H	SNV	Yes	1
DNMT3A	NM_175629	p.N501S	SNV	Yes	1
DNMT3A	NM_175629	p.T275Pfs*41	fms	No	1
ETV6	NM_001987	p.I318Tfs*20	fms	No	1
ETV6	NM_001987	p.I176T	SNV	Yes	1
EZH2	NM_004456	p.G79R	SNV	Yes	1
FBXW7	NM_033632	p.R465C	SNV	Yes	1
FGFR1	NM_001174067	p.L292M	SNV	Yes	1
FLT3	NM_004119	p.I836_M837insG	ins	Yes	1
FLT3	NM_004119	p.I836del	del	Yes	3
FLT3	NM_004119	p.D839G	SNV	Yes	1
FLT3	NM_004119	p.D835V	SNV	Yes	1
FLT3	NM_004119	p.D835Y	SNV	Yes	1
FLT3	NM_004119	p.Y597_E598ins8	ITD24bp	Yes	2
FLT3	NM_004119	p.Q604_F605ins10	ITD30bp	Yes	1
FLT3	NM_004119	p.Y842C	SNV	Yes	1
FLT3	NM_004119	p.D835E	SNV	Yes	1
FLT3	NM_004119	p.N841I	SNV	Yes	1
FLT3	NM_004119	p.M664V	SNV	Yes	1
FLT3	NM_004119	p.N676K	SNV	Yes	1
GATA2	NM_001145661	p.R398T	SNV	Yes	1

GATA2	NM_001145661	p.P470R	SNV	Yes	1
IDH1	NM_005896	p.R132S	SNV	Yes	1
IDH1	NM_005896	p.R119W	MAF<0.01	Yes	1
IDH2	NM_002168	p.R140Q	SNV	Yes	1
IDH2	NM_002168	p.P198A	MAF<0.01	Yes	1
IDH2	NM_002168	p.T435M	MAF<0.01	Yes	1
IDH2	NM_002168	p.R172K	SNV	Yes	1
IKZF1	NM_006060	p.520N*29	fms	Yes	1
IKZF1	NM_006060	p.N159Y	SNV	Yes	1
JAK2	NM_004972	p.V617F	SNV	Yes	4
MPL	NM_005373	p.H624D	SNV	Yes	1
NF1	NM_001042492	p.L2395Ffs*24	fms	Yes	1
NPM1	NM_002520	p.W288Cfs*12	fms	Yes	1
KRAS	NM_033360	p.G12V	SNV	Yes	2
KRAS	NM_033360	p.G12D	SNV	Yes	1
KRAS	NM_033360	p.G12V	SNV	No	1
KRAS	NM_033360	p.L23R	SNV	Yes	1
KRAS	NM_033360	p.G13D	SNV	Yes	1
NRAS	NM_033360	p.G12S	SNV	Yes	1
NRAS	NM_002524	p.G12V	SNV	No	1
NRAS	NM_002524	p.Q61L	SNV	Yes	1
PAX5	NM_016734	p.V26Afs*49	fms	No	1
PAX5	NM_016734	p.C64F	SNV	Yes	1
PAX5	NM_016734	p.P80R	SNV	Yes	1
PHF6	NM_001015877	p.R274N	SNV	Yes	1
PHF6	NM_001015877	p.R335Mfs*15	fms	Yes	1
PTEN	NM_000314	p.A252T	SNV	Yes	1
PTPN11	NM_002834	p.G503R	SNV	Yes	1
RAD21	NM_006265	p.D276G	SNV	Yes	1
RUNX1	NM_001754	p.N264fs	fms	Yes	1
RUNX1	NM_001754	p.F330fs	fms	Yes	1
RUNX1	NM_001754	p.A251Gfs*5	fms	Yes	1
RUNX1	NM_001754	p.D160N	SNV	Yes	1
RUNX1	NM_001754	p.A149fs	SNV	Yes	1
SETBP1	NM_015559	p.H1100R	SNV	Yes	1
SETBP1	NM_015559	p.D868N	SNV	Yes	2
SF3B1	NM_012433	p.R625C	SNV	Yes	1
SRSF2	NM_001195427	p.P95H	SNV	Yes	3
STAG2	NM_001042749	p.D1136Lfs*10	fms	Yes	1
TCF3	NM_003200	p.G385D	SNV	Yes	1
TCF3	NM_003200	p.S514L	MAF<0.01	Yes	1
TCF3	NM_003200	p.S350fs ins 8nt	fms	No	1
TCF3	NM_003200	p.G46R	SNV	Yes	1
TCF3	NM_003200	p.H579R	SNV	Yes	1
TET2	NM_001127208	p.R1167K	SNV	Yes	1
TET2	NM_001127208	p.R1261H	SNV	Yes	1
TET2	NM_001127208	p.V1718L	SNV	Yes	1
TET2	NM_001127208	p.I1897T	SNV	No	1
TET2	NM_001127208	p.V1718L	SNV	Yes	2
TET2	NM_001127208	p.T625I	SNV	Yes	1

TET2	NM_001127208	p.P1335dup	dup	Yes	1
TET2	NM_001127208	p.A707Lfs*44	fms	Yes	1
TP53	NM_000546	p.Y236D	SNV	Yes	1
TP53	NM_000546	p.G245S	SNV	Yes	1
TP53	NM_000546	p.R282P	SNV	Yes	1
TP53	NM_000546	p.A78V	neutral	Yes	1
TP53	NM_000546	p.K132R	SNV	Yes	1
TP53	NM_000546	p.R248N	SNV	Yes	1
TP53	NM_000546	p.V157G	SNV	Yes	1
TP53	NM_000546	p.R123*	fms	No	1
U2AF1	NM_006758	p.S34F	SNV	Yes	1
U2AF1	NM_006758	p.G218V	SNV	No	1
